# Mouse Transplant Models for Evaluating the Oncogenic Risk of a Self-Inactivating XSCID Lentiviral Vector

**DOI:** 10.1371/journal.pone.0062333

**Published:** 2013-04-23

**Authors:** Sheng Zhou, Zhijun Ma, Taihe Lu, Laura Janke, John T. Gray, Brian P. Sorrentino

**Affiliations:** 1 Division of Experimental Hematology, Department of Hematology, St. Jude Children’s Research Hospital, Memphis, Tennessee, United States of America; 2 Veterinary Pathology Core, St. Jude Children’s Research Hospital, Memphis, Tennessee, United States of America; Emory University School of Medicine, United States of America

## Abstract

Hematopoietic stem cell gene therapy requires the use of integrating retroviral vectors in order to stably transmit a therapeutic gene to mature blood cells. Human clinical trials have shown that some vector integration events lead to disrupted regulation of proto-oncogenes resulting in disordered hematopoiesis including T-cell leukemia. Newer vectors have been designed to decrease the incidence of these adverse events but require appropriate pre-clinical assays to demonstrate safety. We have used two distinct mouse serial transplant assays to evaluate the safety of a self-inactivating lentiviral vector intended for use in X-linked severe combined immunodeficiency (XSCID) gene therapy trials. These experiments entailed 28 months of total follow-up and included 386 mice. There were no cases in which the XSCID lentiviral vector clearly caused hematopoietic malignancies, although a single case of B cell malignancy was observed that contained the lentiviral vector as a likely passenger event. In contrast, a SFFV-DsRed γ-retroviral vector resulted in clonal transformation events in multiple secondary recipients. Non-specific pathology not related to vector insertions was noted including T cell leukemias arising from irradiated recipient cells. Overall, this comprehensive study of mouse transplant safety assays demonstrate the relative safety of the XSCID lentiviral vector but also highlight the limitations of these assays.

## Introduction

Stem cell gene therapy is an experimental approach for treating blood disorders in which autologous hematopoietic stem cells are stably transduced in order to introduce a therapeutic gene into the stem cell as well as its progeny. This approach has been studied for the treatment of a variety of immunodeficiency disorders such as X-linked severe combined immunodeficiency (XSCID) [1], [2], ADA-deficiency [3]–[6], Wiskott Aldrich Syndrome (WAS) [7], β-thalassemia [8] and adrenoleukodystrophy [9] and has resulted in significant clinical benefit in these trials. However, in several of these trials, cases have arisen in which the vector has induced T cell leukemia via insertional activation of cellular proto-oncogenes [10]–[13]. The T cell oncogene, LMO2, has been recurrently involved in gene therapy trials for XSCID [13]–[15] and WAS. Efforts are now underway to reduce or eliminate this serious complication and second generation trials with safety-modified vectors are currently underway [16]. A widely utilized approach involves the use of self-inactivating lentiviral vectors that lack enhancer sequences within the vector and include chromatin insulator elements to shield the surrounding chromatin from vector sequences [17]. These vectors are currently being used in clinical trials and may offer a safer approach, particularly for XSCID gene therapy [16].

A current challenge for the field is identifying preclinical assay systems that allow for vector safety tests to be performed prior to implementation of clinical trials [18]–[20]. The risk of T cell leukemia with gene therapy for XSCID was not predicted by independent mouse transplant studies prior to initiation of human clinical trials [21]–[24]. Newer preclinical assays for vector-induced genotoxicity are available and have proven useful but are subject to several limitations. Homologous recombination has been used to recreate LMO2 insertion events found in some human XSCID leukemia cases and allow shuttling different vector elements into the endogenous LMO2 locus to determine relative transactivation potentials [17], [25]. While this is a useful test for XSCID gene therapy, it is limited by being site specific and available in only the T cell lineage. A myeloid immortalization assay has been used to test vector safety and is not necessarily limited to insertion events at one locus, however, most of the immortalizing events occur in a small subset of genes including Evi1 and Prdm16 [26]–[29]. Mouse transplant assays can detect a variety of vector-related hematopoietic transformation events [27], [30] and are not limited by predetermined integration sites. However, mouse transplant studies are expensive, time consuming, and can be relatively insensitive to vector-induced transformation events [19]. The sensitivity can be increased by using tumor prone mouse models which carry a targeted mutation in a tumor suppressor gene [31]–[33], but this approach increases the background rate of tumor formation and decreases the specificity of the assay.

In this study, we have evaluated the safety of a self-inactivating lentiviral vector for XSCID in two different murine, long-term, serial transplant assays. Each of these studies took place over 10 to 14 months and included control vectors that have been previously associated with hematopoietic transformation and clonal skewing of hematopoiesis [30]. The majority of transplanted mice were studied by complete necropsy, flow cytometry analysis, and immunohistochemistry. A complete molecular analysis was performed in all tumor cases associated with the XSCID lentiviral vector. These studies show the advantages and limitations associated with long term murine transplant studies to evaluate vector safety.

## Results

### Design of Vector Safety Assay based on Serial Transplantation in Mice

**Figure 1:**
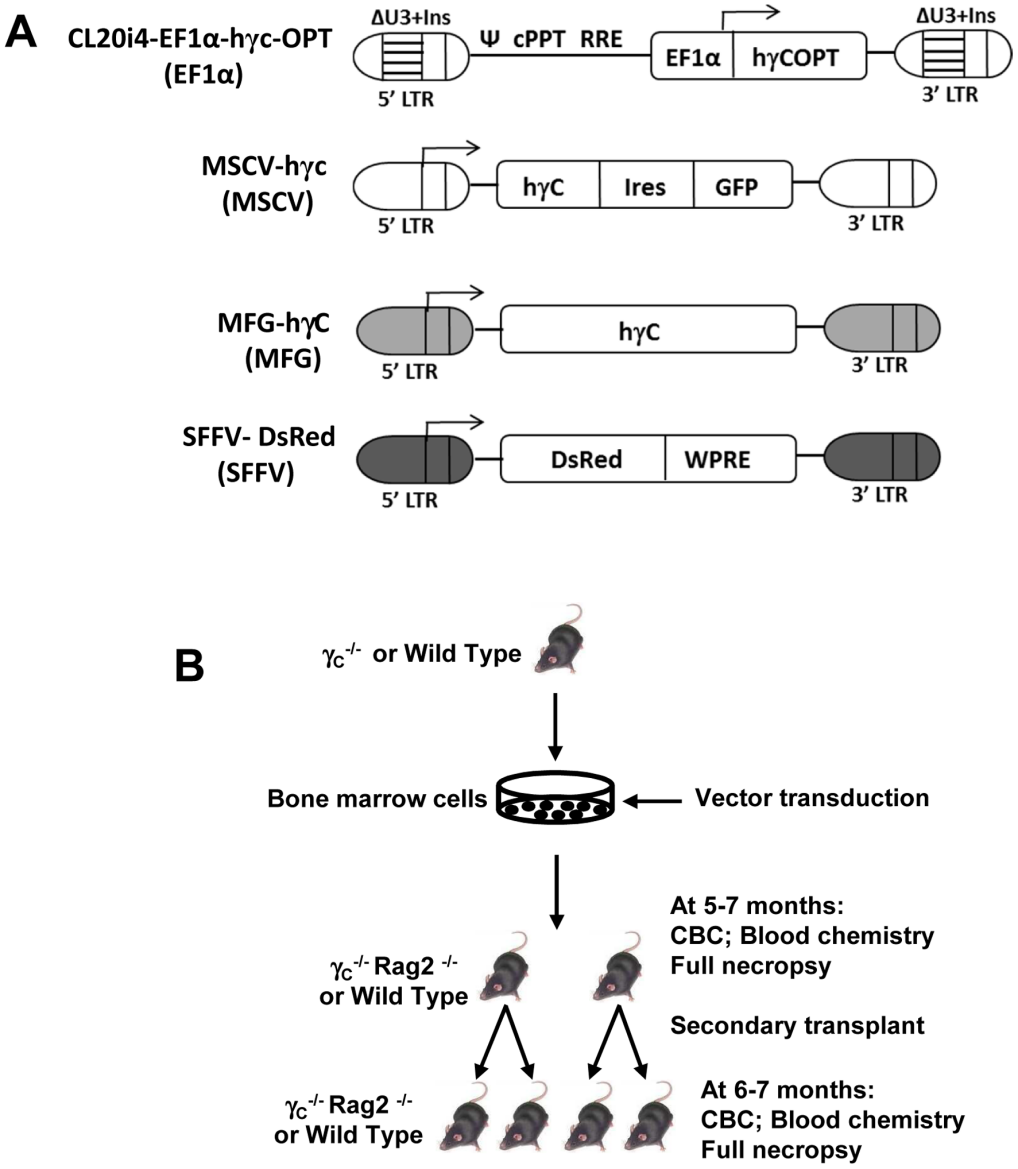
Schematic representation of vectors and mouse transplant experiments. (A) The self-inactivating lentiviral vector for XSCID and three gamma retroviral vectors that were used in this study are shown. The deleted U3 of the HIV-1 LTR was replaced with the 400 bp fragment from chicken beta-globlin insulator element (ΔU3+Ins). The codon optimized human IL2RG cDNA (hγcOPT), the eukaryotic elongation factor 1α promoter (EF1α); the central polypurine tract (cPPT), the Rev response element (RRE) and the psi packaging sequence (Ψ) are also shown. MFG-hγc is the γ- retroviral vector used in XSCID gene therapy trials. A MSCV vector expressing both a human γ_c_ cDNA and an IRES-GRP cassette is shown. The spleen focus-forming virus backbone expressing the DsRed fluorophore is also shown (SFFV-DsRed). Arrows indicate the transcription start sites for each vector. (B)A schematic design of the transplant experiments is shown. Bone marrow cells from γc^−/−^ or normal mice were transduced and were transplanted into lethally irradiated primary recipient mice of either of the indicated genotypes. Five to seven months later, transplanted mice were euthanized and bone marrow cells from each primary recipient were transplanted into two or three secondary recipient mice. The endpoints for these transplant groups are summarized.

The overall goal of this study was to test the safety of the CL20i4-EF1α-hγ_c_-OPT vector [17], which contains a codon-optimized human gamma chain (γ_c_) cDNA under control of a 233 bp version of the cellular elongation factor alpha (EF1α) promoter (Figure 1A). The following γ-retroviral vectors were used as controls: MSCV-hγ_c_-IRES-GFP, MFG-hγ_c_, and SFFV-DsRed (Figure 1A). The MFG-hγ_c_ vector has been associated with LMO2 activation and T cell leukemia in human XSCID trials and the SFFV-DsRed can induce myeloid leukemia in mouse transplant assays [30].

A murine serial transplant assay was used to test the relative safety of these vectors based on prior studies showing that serial transplantation increases the sensitivity for detecting vector-induced hematopoietic transformation [30]. In the first of two experiments, bone marrow cells from Il2rg^−/−^ mice were transduced and then transplanted into sublethally irradiated Rag2^−/−^, γ_c_^−/−^ recipient mice which were then observed for 20 to 28 weeks after transplant (Figure 1B). At the end of the observation period, bone marrow cells were collected from each primary recipient and used to transplant two to three secondary Rag2^−/−^, γ_c_^−/−^ recipients that were followed for an additional 24 to 28 weeks. The experiment was concluded by full necropsy and hematologic analysis of all the secondary recipients. A second experiment was performed using wild type C57BL/6J mice as donors and recipients. Recipient mice were lethally irradiated and observed 20 to 28 weeks after transplantation.

### Pathology not Related to Retroviral Vectors

In experiment 1, fifteen primary recipients were transplanted with cells transduced with the EF1α lentiviral vector or with the MSCV γ-retroviral vector, and 11 were transplanted with mock-transduced cells (Table 1). No mice from any of these groups developed hematological malignancies or other toxicities related to the vector. Secondary transplants were performed using 23 secondary recipients for the mock group, 36 for the MSCV group, and 39 recipients for the EF1α group (Table 2). Significant but partial immune reconstitution was seen in secondary recipients as evidenced by an increase in T cell numbers in the peripheral blood (Figure S1) and of T, B, and NK cell numbers in the spleen (Figure S2).

**Table 1:** Malignancy incidence in primary recipient mice in Exp. 1 (donor: γ_C_^−/−^, recipient: γ_C_^−/−^ Rag2^−/−^ ).

Vector	# Transplanted	VCN	# Vector+hematologicalMalignancies	# Other death*	# tumor free at autopsy(5–8 months)
Mock	11		0	0	11
MSCV	15	1.4±0.58	0	1	14
EF1α	15	2.17±0.74	0	0	15

**Table 2:** Malignancy incidence in secondary recipient mice in Exp. 1 (donor: γ_C_^−/−^, recipient: γ_C_^−/−^ Rag2^−/−^ ).

Vector	# Primary donor mice for secondary transplant	# Secondary recipients	# Vector+ Hematological Malignancies	# Other death*	# tumor free at autopsy(6–7 months)
Mock	11	23	0	1	22
MSCV	14	36	0	13	23
EF1α	15	39	**3** **	3	33

* Other death: Mice died or sacrificed before reaching end points, but not due to hematological malignancies that have transgene insertion (for examples, recipient derived tumor).

** All three mice are from the same donor.

VCN: Vector copy number in peripheral blood mononuclear cells at 30 weeks.

Of all 98 secondary recipients, 17 mice died due to non-specific causes unrelated to the vector. For example, one mouse in the mock group died 24 weeks after transplantation due to severe anemia and was found to have a malignant granulosa cell tumor of the ovary with secondary hemorrhage. Thirteen mice in the MSCV group did not reach the end point and the cause of death or sickness could not be identified in most cases, however; hematologic malignancies were not found on autopsy in any of these cases. Three mice in the EF1α group died from non-specific causes including acidophil macrophage pneumonia, pyogranulomatous myocarditis, and autopsy findings were consistent with systemic infections.

### A Single Case of B-cell Lymphoma Associated with the EF1α Vector

**Figure 2:**
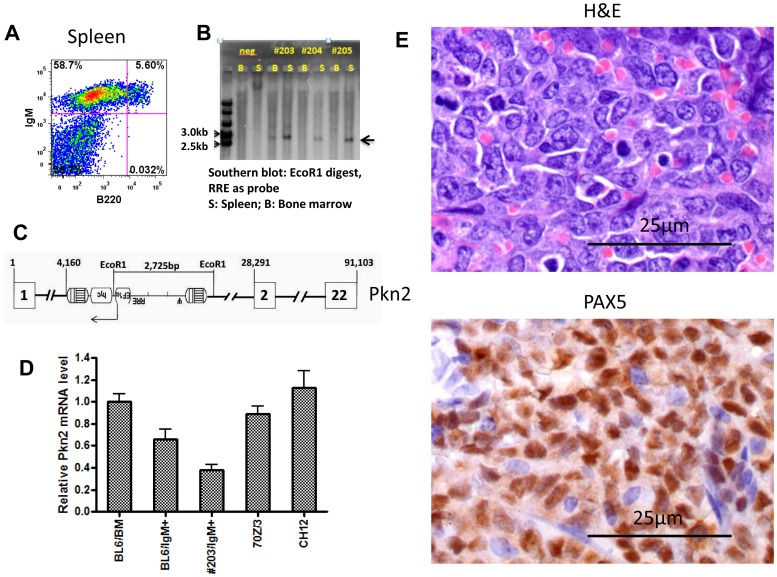
Characterization of B cell leukemias in three secondary recipients that received bone marrow cells from one primary recipient in experiment 1 in the EF1α vector group. (A) Flow cytometry analysis of spleen cells in an affected secondary recipient. Staining was done for surface expression of IgM (Y axis) and B220 (X axis) and percentages of cells in each gate are indicated. (B) Southern blot analysis of DNA from bone marrow and spleen cells using a vector probe and a single cutter enzyme for insertion site analysis. Bone marrow and spleen cell samples are indicated and identification of individual secondary mice is shown. The arrow indicated a clonal band derived from a single vector insertion in all three cases. (C) Inverse-PCR identification of the vector insertion event is shown. The vector is inserted in the first intron of the Pkn2 gene in the reverse genomic orientation. The location of the EcoRI sites used in the Southern analysis is shown as well as relative nucleotide distances. Exons are shown in boxes with corresponding numbers. (D) Quantitative real time PCR analysis of the Pkn2 transcripts in tumor cells and controls. Unfractionated total bone marrow cells (BL6/BM) was used as Pkn2 expression reference and the value was arbitrarily set as 1. Also shown are values for sorted IgM+ cells from spleen of a healthy C57BL6 mouse (BL6-IgM), sorted IgM+ leukemic cells from spleen of secondary recipient mouse (#203/IgM^+^), murine pre-B cell lymphoma cell line (70Z/3), and a murine mature-B cell lymphoma cell line (CH12). (E) Histology and immunohistochemistry for Pax5 expression are shown for a splenic B cell tumor from a secondary recipient are shown with size markers in the lower left of each panel.

All the three secondary recipient mice in the EF1α group (#203, #204, #205), which all received bone marrow cells from a single primary donor (#364), developed a B cell malignancy at five to six months after transplant. All three mice had enlarged spleens containing an abnormal IgM^+^/B220^−^ cell population comprising approximately 60% of all spleen cells (Figure 2A). Pathologic analysis showed infiltration of lymphoma cells in spleens of these mice and these tumor cells expressed Pax5, a B cell specific transcription factor (Figure 2E). Mouse #203 also had infiltration of lymphoma cells in the heart, lung and liver (data not shown). The lymphoma cells from all three mice have a single vector insertion as shown in the Sounthern blot (Figure 2B). Vector integration analysis was performed using inverse-PCR to clone out the genomic junction sequences flanking the lentiviral LTR. These genomic sequences mapped to intron 1 of *Pkn2* and the vector was inserted in an antisense orientation (Figure 2C). To confirm these mapping results, Southern analysis showed the expected 2.7 kb EcoRI fragment in spleen samples from all three secondary mice (Figure 2B) demonstrating that the B cell malignancy is derived from the same clone in all three cases and that the tumor initiating cell arose in the primary recipient. The next closest known gene (Gtf2b) from the insertion site is 94.2 kb away.

Pkn2 is a serine/threonine protein kinase, is a target of Rac and Rho GTPases, is a mediator of a variety of cell signals, and has been implicated in tumor cell migration and invasion [34]. To determine whether this insertion event resulted in altered *Pkn2* expression, IgM^+^ cells from the spleen of mouse #203 were isolated by cell sorting and qRT-PCR was used to measure Pkn2 mRNA expression (Figure 2D). Pkn2 expression in tumor cells was compared to that seen in normal bone marrow cells, in sorted IgM^+^ cells from a spleen of a healthy C57BL6 mouse, to the mouse pre-B cell lymphoma cell line (70Z/3), and to the mouse mature B cell lymphoma cell line (CH12). The leukemic cells from mouse #203 expressed about 50% less Pnk2 mRNA than that seen in normal bone marrow and spleen cells (Figure 2D). The Pnk2 mRNA level was also lower than that seen in either of the two B cell lymphoma cell lines. These results demonstrate that the vector insertion event did not result in transactivation of Pkn2 but may have led to mono-allelic gene inactivation. Another possibility is that this relative low expression simply reflects the lack of proper references for this measurement, and Pkn2 expression is intrinsically low in these lymphoma cells.

### Transformation of Wildtype Bone Marrow Cells with the SFFV Vector

In experiment 2, bone marrow cells from B6.SJL-*Ptprc^a^ Pepc^b^*/BoyJ mice were transduced either with the MFG, SFFV, EF1α vector, or under mock conditions, and then transplanted into 15 to 19 primary recipients per group. Efficient transduction of hematopoietic stem cells was achieved in recipient mice in all groups as demonstrated by vector copy number analysis (Table 3). Peripheral blood counts and serum chemistries were normal and did not differ between mock transduced and vector transduced groups (Figures S3 and S4). One mouse in the MFG-γ_c_ group developed a large keratoacanthoma in right hind leg at 24 weeks. Another mouse in the SFFV-DsRed group (#8053) also developed a large keratoacanthoma requiring euthanasia at 22 weeks. None of the primary transplant mice in any group showed any signs of hematological malignancies.

**Table 3:** Malignancy incidence in primary recipient mice in Exp 2 (Donor: CD45.1^+^; Recipient: CD45.2^+^).

Vector	# Transplanted	VCN	# Vector+hematologicalMalignancies	# Other death*	# tumor free at autopsy(7–8 months)
Mock	15		0	1	14
MFG	15	0.81±0.31	0	1	14
SFFV	19	1.81±1.23	0	2	17
EF1α	15	1.59±0.39	0	0	15

**Figure 3:**
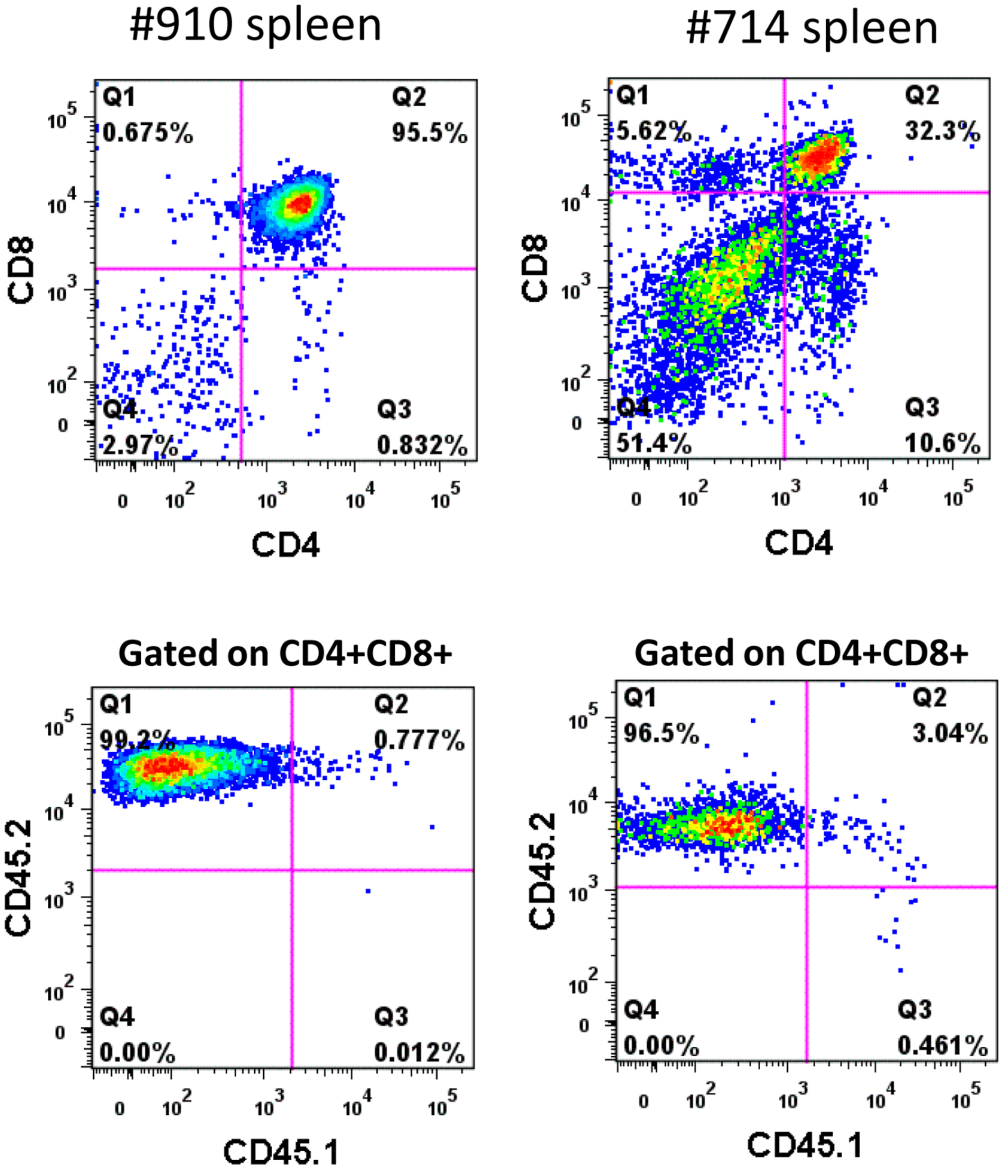
Recipient origin of T-cell malignancies arising in the secondary recipients of the EF1a group in experiment 2. Tumor cells derived from the spleen of transplanted mice were analyzed for CD4 and CD8 expression first (top panels). The abnormal CD4^+^ CD8^+^ leukemic cells were then gated and analyzed for CD45.1 (donor) and CD45.2 (recipient) marker expression. Virtually all CD4^+^CD8^+^ cells exclusively expressed CD45.2 and were therefore derived from the irradiated recipient mice.

A total of 183 C57Bl/6J mice were used as secondary recipients with three secondary transplant mice set up for each primary donor (Table 4). Recipient-derived hematologic malignancies were noted in three mice from the mock group, in three from the EF1α group, and in one from the SFFV group. The donor cells used in the primary transplant were derived from B6.SJL-*Ptprc^a^ Pepc^b^*/BoyJ mice expressing the CD45.1^+^ allele while CD45.2^+^ mice were used as transplant recipients. One secondary recipient from the mock group developed a CD4^+^ CD8^+^ mediastinal lymphoma that was CD45.2^+^ and therefore was derived from endogenous hematopoietic cells remaining in the recipient after irradiation. In the EF1α group, three secondary mice developed T-cell leukemia with enlarged spleen and thymus (Table S1). The leukemic population in the spleen was comprised of CD4^+^CD8^+^ T-ALL cells and exclusively expressed the CD45.2^+^ recipient marker (Figure 3). These results demonstrate the potential for background hematopoietic malignancies to arise from recipient-derived cells and the importance of distinguishing the donor from the recipient background.

**Table 4:** Malignancy incidence in secondary recipient mice in Exp 2 (Donor: CD45.1^+^; Recipient: CD45.2^+^).

Vector	# Primary donormice for secondarytransplant	# Secondary recipients	# Vector+HematologicalMalignancies	# Recipient derived hematological malignancies	# Other death*	# tumor free at autopsy(6–7 months)
Mock	14	42	0	3	2	37
MFG	14	42	1**	0	2	39
SFFV	18	54	**7** ***	1	2	44
EF1α	15	45	0	3	2	40

* Other death: Mice died or sacrificed before reaching end points, but not due to hematological malignancies.

** not definitive in terms of vector presence.

*** P<0.05.

VCN: Vector copy number in peripheral blood mononuclear cells at 30 weeks.

**Figure 4:**
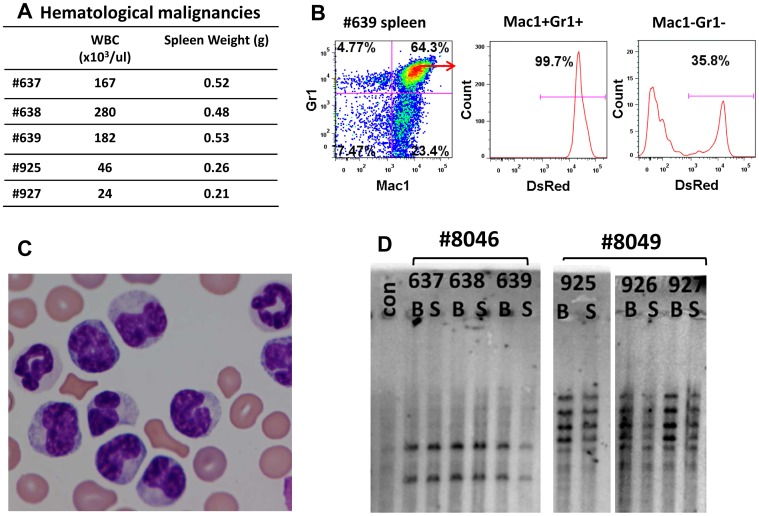
Characterization of myeloid malignancies seen in the SFFV-treated secondary recipients. (A) Hematologic characteristics of myeloid leukemias including peripheral leukocyte counts and spleen weights in individual mice at the time of euthanasia. (B)Flow cytometry analysis of splenic tumor cells from mouse #639. Staining for the Gr1 and Mac1 myeloid markers is shown. Gated normal cells (Gr1^−^ Mac1^−^ ) and tumor cells (Gr1^+^ Mac1^+^) were also analyzed for expression of the DsRed, vector-encoded marker. (C) Peripheral blood smear from a leukemic mouse with abnormal monocytic and granulocytic cells. (D) Southern blot analysis of DNA from bone marrow (B) and spleen (S) of six secondary mice that were derived from two primary recipients is shown. A clonal analysis for vector insertion sites was performed using a single cutter enzyme (BglII) and probed with DsRed cDNA probe. A common clonal pattern was noted from tumors derived from each primary recipient. All mice but #926 had clinical evidence of leukemia, however it was noted that #926 had already converted to a clonal pattern.

Seven cases of vector-induced malignancies occurred in the SFFV vector group. Myeloid leukemia was detected in secondary recipients #637, #638, and #639, all of which were transplanted with donor cells derived from the same primary recipient mouse (#8046). Similarly, myeloid leukemia occurred in secondary recipients #925 and #927, which received donor cells from the same primary recipient mouse #8049. All five mice had enlarged spleens and elevated peripheral leukocyte counts ranging from 24 to 280×10^3^ cells/ul (Figure 4A). The tumors expressed the vector-encoded DsRed gene and the Gr1 and Mac1 myeloid markers (Figure 4B). The morphology of tumor cells in the peripheral blood showed various stages of monocytic and myeloid differentiation (Figure 4C). Southern analysis for vector integration sites demonstrated that tumors in secondary mice were clonal and originated in the primary recipient (Figure 4D). In cases 637, 638, and 639, two vector integrations were present in the bone marrow and spleen of all cases. In cases 925 and 927, there were 5 independent insertion sites noted in both cases. Inverse-PCR showed that one of these insertions was in the Mecom gene, also known as Evi-1 (Figure S5). Two additional secondary recipients in the SFFV group presented with a B-cell leukemia that expressed the DsRed marker and were derived from the same primary donor.

### Myeloid Skewing and Immortalization with the SFFV-DsRed Vector

**Figure 5:**
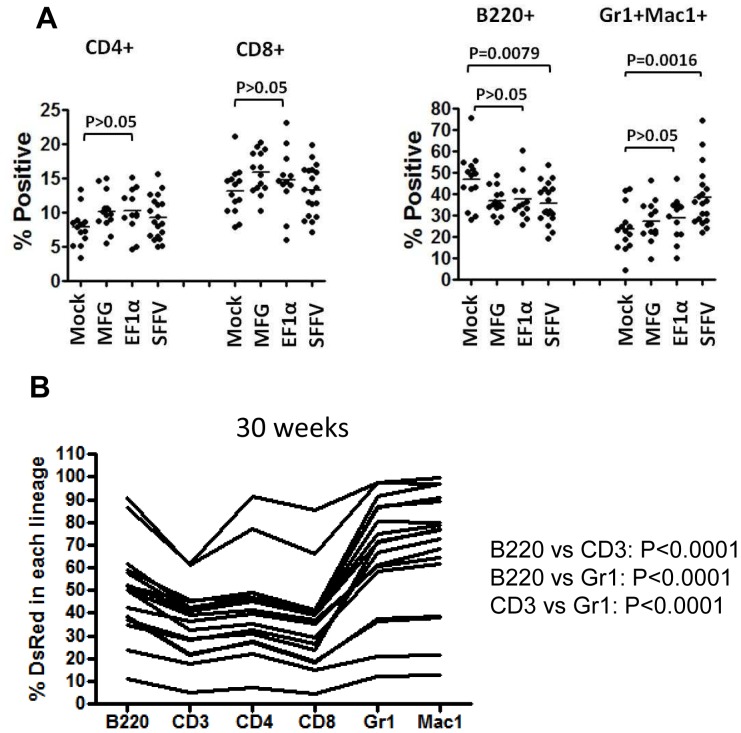
Skewing of myeloid differentiation in mice transplanted with SFFV-transduced cells. (A) Percentage of peripheral blood cells in specific hematopoietic lineages at 30 weeks after transplantation. Each dot represents the percent of cells from a given lineage, as determined by flow cytometry, from a single primary transplant recipient from each of the vector groups listed on the X-axis. Statistical comparisons are shown for the indicated groups giving the p value. This analysis demonstrated a significant increase in cells expressing either the Gr1 or the Mac1 marker. (B) Percentage of DsRed+ peripheral blood cells in each lineage in the SFFV group of primary recipients at 30 weeks after transplant. Each line represents the profile from a single mouse and the lineages are indicated on the X-axis.

Analysis of the lineage distribution of peripheral blood cells in primary recipient mice showed a significant increase in the proportion of Gr1^+^ and/or Mac1^+^ myeloid cells in the SFFV vector group (Figure 5A). Expression of the vector-encoded DsRed reporter gene also occurred in a significantly higher proportion of myeloid cells versus the B or T cells at 30 weeks after transplant (Figure 5B). These results suggest that myeloid skewing associated with vector expression may be an early and sensitive surrogate endpoint for insertional transformation in this model.

Myeloid transformation has been associated with SFFV integration in an in vitro myeloid immortalization assay [26], which was used to compare the transformation potential of the SFFV vector with the MFG and the EF1α vector in three independent transductions. A relatively high number of immortalized clones was associated with the SFFV vector at copy numbers ranging from 1.67 to 3.35 vector copies per genome (Table 5, Figure S6). The lower vector copy number in the higher MOI SFFV group could be due to interference of vector preloading when highly concentrated vector was used. In contrast, no immortalization activity was seen with the EF1α vector or with mock-transduced cells. A single immortalized clone was observed using the MFG vector at an MOI of 30. These results provide similar information to that obtained in the second transplant model and show the relative lack of transforming potential associated with the EF1α lentiviral vector.

**Table 5:** Myeloid immortalization assay.

	Positive wells(100 cells/well)	VCN
Mock-1	0	0
Mock-2	0	0
Mock-3	0	0
SFFV-1 (MOI = 10)	92	3.3
SFFV-2	75	3.35
SFFV-3	96	3.26
SFFV-M 1 (MOI = 20)	2	1.95
SFFV-M2	0	1.87
SFFV-M3	96	1.86
SFFV-H1 (MOI = 30)	0	1.67
SFFV-H2	96	1.73
SFFV-H3	76	1.83
MFG -1 (MOI = 30)	0	3.7
MFG-2	0	2.8
MGF-3	1	3.49
EF1a-1 (MOI = 30)	0	1.49
EF1a-2	0	1.54
EF1a-3	0	1.76

## Discussion

The repeated occurrences of hematologic malignancies related to stem cell gene therapy mandate the development of effective preclinical assay systems to estimate the transforming potentials associated with different vectors and transduction protocols. Mouse models of retroviral stem cell gene therapy offer several advantages including the lack of any predetermined assumptions about which proto-oncogenes or specific insertion events are most relevant and the availability of human disease models arising from gene targeting in mouse embryonic stem cells. The XSCID background is known to be a predisposing factor to vector-induced T cell leukemia [32], [35], [36], potentially due to the potent clonal selection and expansion associated with corrected lymphocyte progenitors and alterations in the biologic status of target cells in the XSCID bone marrow [32], [37], and the possibility that arrested T cell progenitors may accumulate collaborating mutations associated with Lmo2-induced transformation [13], . However, we found that the XSCID background was associated with a relatively high rate of non-specific pathology, particularly in the secondary recipients, making it more difficult to determine which adverse events were directly related to vector insertion events.

Three cases of B-cell lymphoma occurred in secondary recipients in which the EF1α lentiviral vector was present in the tumor cell genome. All three cases originated from a single transformed clone that was present subclinically in a single primary recipient illustrating the importance of using distinct secondary recipients for each primary transplanted donor in order to quantitate transformation frequencies. The EF1α lentiviral vector was integrated into the *Pkn2* gene raising the question of whether this event was an actual driver of transformation. Over-expression of Pkn2 has been associated with malignant progression [34]; however, in this case, Pkn2 expression was decreased by about 50%, possibly due to inactivation of one allele by the vector insertion. The likelihood that the *Pkn2* insertion was a passenger event is further supported by the fact that B-cell malignancies arise spontaneously in approximately 50% of mice at 2 years of age [39], [40]. The results show that the mere presence of a vector integration event in a tumor clone within or in the vicinity of a potential proto-oncogene does not necessarily invoke a functional role for that insertion in oncogenesis.

A second model was based on transplant of normal bone marrow cells from C57Bl/6J mice and was associated with less non-specific pathology compared to the XSCID model, most likely due to the lack of the immunodeficiency background and attendant complications. Recipient-derived T cell malignancies arose in seven out of 183 secondary recipients, likely due to radiation-induced transformation of residual thymocytes [41]. The occurrence of these vector-independent T cell malignancies underscores the importance of using specific markers that distinguish donor versus recipient background.

In addition, seven cases of hematopoietic malignancy were observed in secondary recipients that were likely due to SFFV-induced insertional mutagenesis. Five of these cases were clonal myeloid malignancies derived from two primary cases and exhibited the features of SFFV-induced myeloid malignancies (Table S1) including the typical leukemogenic Evi-1 gene insertion [27]. No tumors were seen with the EF1α lentiviral vector at a similar vector copy number to that seen with the SFFV vector. This comparison demonstrates the relative safety of the EF1α lentiviral vector in this myeloid transformation assay system; however, only a relatively small number of vector-related transformation events were seen with the positive control vectors. It may be possible to increase the sensitivity of this assay by using skewed myeloid differentiation as a surrogate endpoint rather than malignancy per se. Primary recipients transplanted with SFFV-transduced cells showed an increased proportion of Gr1^+^ Mac1^+^ cells in the peripheral blood and a significantly increased proportion of vector-expressing cells in the myeloid lineages at 30 weeks after transplant. However, the detection of these endpoints was facilitated by a distinct flow cytometry marker present in the SFFV vector, a feature that is lacking in most therapeutic vectors.

Neither of the mouse transplant assays nor the in vitro immortalization assay has resulted in Lmo2 activation events such as those that have recurred repeatedly in XSCID human gene therapy trials [13], [15]. The reason for the lack of Lmo2 integrations in murine cell based assays is currently unknown and may be due to intrinsic differences between CD34-selected human hematopoietic cells and unsorted or lineage negative mouse bone marrow repopulating cells.

Clinical gene transfer using retroviral vectors for hematological disorders typically involves isolation of CD34+ cells, transduction of the cells with the vectors in the presence of recombinant cytokines, such as throbopoietin (TPO), stem cell factor (SCF), Fms-like Tyrosine Kinase-3 ligand (FLT3L) and interleukin-3, and transfusion of the cells back to patients. Direct assessment of the insertional oncogenesis activity of vectors in the transduced CD34+ cells, ether with in vitro or with in vivo xenograft models, could be very insensitive due to intrinsic low transformation susceptibility of human cells [42]. Given that the majority of the oncogenic events that occurred in the clinical gene transfer were caused by insertional activation of oncogenes mediated by the enhancer elements that are present in the vectors [13], [15], the most relevant assay for this activity is probably the LMO2 activation assay in human Jurkat T lymphocytes that was developed by Ryu et al [25].

Donor cell population and culture conditions may also affect the sensitivity of our transplant assay. While human hematopoietic stem cells are mostly CD34+, the mouse hematopoietic stem cells are enriched in the CD34- subpopulation [43]. Since our study involves both gammaretroviral and lentiviral vectors, and it is known that gammaretroviral vector only transduces proliferating cells, we used 5-FU to treat donor mice before harvesting bone marrow cells in order to enhance the transduction efficiency of the gammaretroviral SFFV and MFG-rc vectors. This treatment may have eliminated some progenitor cells that could potentially be cell-of-origin of the malignancies that develop after transplant. In addition to the use of SCF and TPO, we have also used IGF-II and FGF-I, in order to maintain the hematopoietic stem cells in an undifferentiated stage [44]. This condition may provide selective growth advantage for certain cell populations. The lack of leukemias in the MFG-rc vector group could be due to these reasons. Nevertheless, 7 cases of leukemias occurred with the SFFV vector, which also caused clinical leukemia in the CGD clinical study due to insertional activation of the MDS1-Evi1 gene [29].

In conclusion, we have used long-term mouse transplant assays to test the safety of a self-inactivating lentiviral vector intended for use in XSCID gene therapy. These experiments have supported the relative safety of this self-inactivating lentiviral vector in several assay systems. However, while demonstrably responsive to SFFV-induced transformation, these transplant assays may have limited sensitivity to less oncogenic vectors and subject to a substantial degree of background pathology. Furthermore, these assays are lengthy and costly. Our assays included 386 mice and took 28 months. Newer assays are needed for vector testing that are more expedient, that recapitulate the specific targeting events that have been seen in human clinical trials, and are of greater relevance to gene therapy of XSCID and other immunodeficiencies involving T and B lymphocytes.

## Materials and Methods

### Vectors and Cell Lines

The generation and activity of the lentiviral CL20i4-EF1a-hγc-OPT (also referred as the EF1α vector) has been described before [17], [45]. The gammaretroviral MFG-γc (MFG) vector was kindly provided by Dr. John Gray at St Jude Children’s Research Hospital. The gammaretroviral SFFV-DsRed (SFFV) vector was kindly provided by Dr. Christopher Baum and has been previously described [26]. All these vectors were pseudotyped with a vesicular stomatitis virus G (VSV-G) protein and were transiently produced in 293T cells by cotransfecting with plasmids expressing VSV-g, Gag-Pol (lentiviral or gammaretroviral) and Rev-Tat (for lentiviral vector only). The titer of the EF1a and MFG-γc vector were estimated by transducing ED7R human T leukemia cells or Hela cells and subsequent measurement of cell surface γc expression using an anti-CD132 antibody (Beckton Dickinson, Franklin Lakes, NJ) by flow cytometry. The titer of the SFFV vectors were estimated by transducing Hela cells and subsequent measurement of DsRed or GFP by flow cytometry. The murine pre-B cell lymphoma cell line (70Z/3) [46] and the murine mature-B cell lymphoma cell line (CH12) [47] were maintained in RPMI1640 medium containing 10% fetal bovine serum.

### Mice

C57BL/6J mice expressing CD45.2 antigen and B6.SJL-*Ptprc^a^ Pepc^b^*/BoyJ mice Expressing CD45.1 antigen on white blood cells were purchased from Jackson laboratory. The Il2rg^−/−^ Rag2^−/−^ mice were purchased from Taconic Farms (Hudson, NY). All experimental procedures were reviewed and approved by the Institutional Animal Care and Use Committee of St Jude Children’s Research Hospital.

### Vector Transduction and Bone Marrow Transplantation

The vector transduction and bone marrow transplantation conditions using Il2rg^−/−^ donor cells in experiment 1 have been previously described [17]. For the transplant of normal bone marrow cells into irradiated recipient mice in experiment 2, donor mice were wild type B6.SJL-*Ptprc^a^ Pepc^b^*/BoyJ mice, used at 6–12 weeks old. Recipient mice were wild type female C57BL/6J mice. All mice were obtained from the Jackson Laboratory.

Donor mice were injected with 5-fluorouracil (150mg/kg body weight, intraperitoneally) on day −5. On day 0, mice were killed by carbon dioxide inhalation and bone marrows from both tibia and femurs were harvested into PBS with 2% fetal bovine serum (PBS/2%FBS). Bone marrow cells from all donor mice were combined. Mononuclear cells were cultured overnight in StemSpan SFEM at a concentration of 3×10^6^ cells/ml in the presence of rmSCF, rmTPO, rmIGF-II (all 20 ng/ml), rhFGF1 (10 ng/ml), Heparin (10 ug/ml), penicillin and streptomycin in an incubator with 5% CO2. On day 1, cells were collected and counted and divided into four groups. Group 1 was not transduced with vector and served as a mock control, group 2 was transduced with the MFG-γ_c_ vector (MOI = 4.3), group 3 was transduced with the CL20i4-EF1a-hγc-OPT vector (MOI = 40), and group 4 was transduced with the SFFV-DsRed vector (MOI = 13). Transductions were performed on retronectin-coated tissue culture plates. On day 2, cells were collected, counted, and resuspended into phosphate buffered saline containing 2%FBS. Recipient wild type mice were lethally irradiated (1100rad) with a Cs137 irradiator 1–3 hours before transplantation. Cells were injected into the recipient mice through lateral tail vein. Each recipient mouse received 1–1.5×10^6^ cells (this included transduced and non-transduced cells) in a total volume of 0.5 ml. Mice were monitored daily for activity and sickness. Starting at 12 weeks after transplant, we obtained periodic complete blood counts (CBCs) and flow cytometry analyses of peripheral blood for Gr1+, Mac1+, CD4^+^, CD8^+^, and B220^+^ cell subpopulations at 14 and 30 weeks. Peripheral blood cells were collected on week 30, and genomic DNA was extracted and vector copy number was measured by quantitative PCR method described below. When mice were found to be ill (hunching, limited mobility, enlarged abdomen, obvious mass etc), or were found to have an abnormal elevation in the white blood cell count (>16,000 white blood cells/ul blood), or had reached a predefined end point (greater than 6–7 months after transplantation), end point analyses were performed including complete necropsy by a veterinary pathologist.

### Secondary Transplantation

Seven to eight months after transplantation, all primary recipient mice were euthanized and end point analysis and secondary transplantation were performed. For each primary recipient, 4–5×10^6^ bone marrow cells were used to transplant each of three secondary lethally irradiated recipient mice (1100rad). Bone marrow cells from individual primary mice were not mixed prior to transplant so that each secondary recipient received cells from an individual primary recipient mouse. This was done to allow for better quantification of the transformation rate. The secondary recipient mice were also monitored daily after transplant. If a mouse is found moribund, or with an elevated peripheral leukocyte count (>16,000 white blood cells/ul blood) or has reached a predefined end point (greater than 6 months after transplantation), these mice were sacrificed and end point analyses was performed.

### End Point Analysis

CBC and blood chemistry were measured using automated equipment in our veterinary facility, blood smears were prepared and microscopically reviewed, and full necropsy performed. The full necropsy included gross visual inspection of all organs; documentation of organomegaly; formalin fixation, paraffin embedding, sectioning and H&E staining of samples from all major organs. In all animals, the following organs/tissues were examined: heart, skeletal muscle, tongue, thyroid glands, lungs, esophagus, liver, kidneys, adrenal glands, thymus, spleen, salivary glands, stomach, small intestine, cecum, colon, pancreas, reproductive organs, skin, brain (cerebellum and cerebrum), pituitary glands, eyes, harderian glands, ears, bone marrow; For each animal, only organs in which lesions were present are described in this report; the organs not specifically mentioned were without lesions and appeared normal. If lesions, except malignancies, were present in all three or 4 vector groups, these lesions are considered non-specific and not specifically caused by the CL20i4-EF1a-hγc-OPT vector. If a mouse had an enlarged spleen, thymus or lymph nodes, and had a clonal malignant cell population in these organs and bone marrow, this mouse was then diagnosed as having hematological malignancy. The nature of the malignancies was then studied by immune-phenotyping of the malignant cell population by flow cytometry and/or immunohistochemistry. Lesions in other organs were carefully examined in histology to determine if it was a metastasis from a hematological malignancy.

If either the spleen or thymus or both were enlarged, cells from the thymus, spleen and bone marrow were analyzed in flow cytometry for lineage and vector expression using at least the following markers(CD4+, CD8+, Gr1+, Mac1+, B220+, IgM+, DeRed) (all antibodies were from Beckton Dickinson). Splenic cells and bone marrow cells were also used to extract genomic DNA for vector integration site and copy number analysis.

### Vector Copy Number Analysis

Genomic DNA from peripheral blood mononuclear cells of recipient mice were extracted for vector copy number assessment by a quantitative PCR analysis using relative standard curve quantitation method with StepOnePlus instrument and software (Life Technologies, Carlsbad, CA). Primers/probe sequences specific for the lentiviral vector backbone, the GFP gene, the MFG vector backbone and the WPRE element of the SFFV vector were derived. Assay for internal control for mouse cells is beta-actin (#4352341E, Life Technologies, Carlsbad, CA,). Genomic DNA from mouse leukemic cells with single copy integration of the lentiviral backbone containing GFP was used to generate standard curves for lentiviral vector and vectors containing GFP. Serial dilutions of MFG vector plasmid DNA into mouse genomic DNA was used to generate standard curve for the MFG vector. Genomic DNA from NIH3T3 cells containing a single copy SFFV-GFP vector insertion was used to generate standard curve for the SFFV vector, which contains the WPRE element. The primer and probe sequences used for quantification of vector copy number are as follows:

For the lentiviral vector, as described in [48]:

LentiGAG.probe 5′-ACAGCCTTCTGATGTTTCTAACAGGCCAGG-3′

LentiGAG.forward 5′-GGAGCTAGAACGATTCGCAGTTA-3′

LentiGAG.reverse 5′-GGTTGTAGCTGTCCCAGTATTTGTC-3′

For GFP, as described in [49]:

EGFP.probe: 5′- AAAGACCCCAACGAGAAGCGCGA -3

EGFP-F: 5′-CTGCTGCCCGACAACCA-3′

EGFP-R: 5′-GAACTCCAGCAGGACCATGTG-3′

For MFG-γ_c_ vector backbone, as described in [50]:

MFG.probe: TACCCGTGTATCCAATAAACCCTCTTGCAGTT

MFG-F: ATTGACTGAGTCGCCCGG

MFG-R: AGCGAGACCACAAGTCGGAT

For WPRE element of the SFFV vectors:

WPRE.probe: TGGTGTGCTCTGTGTTTGCTGAC

WPRE-F: TCCTGGTTGCTGTCTCTTTATG

WPRE-R: CAGAAAGGAGTTGACAGGTGG

### Flow Cytometry Analysis for Reconstitution

Peripheral blood was collected and red blood cells were lysed with a red blood cell lysis buffer before antibody labeling. Cells from bone marrow, spleen and thymus were used directly for antibody labeling. Cells were labeled with lineage antibodies-specific conjugated with different fluorophores (CD3, CD4, CD8, B220, Gr1, Mac1, CD45.1, CD45.2) and then analyzed in a LSR flow cytometer (Becton_Dickinson, Franklin Lakes, NJ). The data were analyzed using Flowjo software (Tree Star, Ashland, OR).

### Identification of Vector Insertion Sites

Provirus insertion sites in the leukemias were identified using an inverse-PCR method. Genomic DNA from bone marrow or spleen of mice that developed leukemia were extracted and digested with Fat1 restriction endonuclease. After ligation, the DNA template was subject to PCR (94°C for 1 min, 58°C for 1 min, 72°C for 3 min, 40 cycles) with primers 5′ GGGACAGCAGAAATCCACTTTG 3′ and 5′ GATCGCTTTCCTCTGAAC 3′. The final PCR product were directly sequenced or sequenced after subcloning into a vector.

### qRT-PCR for Measuring Expression of Pkn2 in Tumor Sample

Total RNAs were extracted from sorted leukemic IgM+ B cells from bone marrow of the leukemic mouse (#203); from murine pre-B lymphoma cell line 70Z; from murine mature B cell lymphoma cell line CH12.1.16; from sorted IgM+ B cells and total bone marrow cells of a healthy C56BL/6J mouse. The total RNAs were then subject to reverse transcription using the SuperScript VILO cDNA Synthesis Kit (Life Technologies, Grand Island NY). The expression of Pkn2 gene was then measured using QuantiTect Primer Assays (#QT00153251 for Pkn2, and #QT01136772 for beta-actin, Qiagen, Valencia, CA) and QuantiTect SYBR Green PCR Master Mix (Qiagen, Valencia, CA).

### Myeloid Immortalization Assay

We essentially followed the protocol developed by Modlich et al for the myeloid immortalization assay [14]. Lineage-negative (Lin^-^) BM cells were isolated from 5 female C57BL6/J mice (6–8 weeks old, Jackson Laboratory) using Lineage Cell Depletion Kit (Miltenyi, Auburn CA). Lin^–^ BM cells were prestimulated for 2 days in Iscove’s Modified Dulbecco’s Medium (Invitrogen) containing 50 ng/ml recombinant murine stem cell factor, 100 ng/ml recombinant human Flt-3 ligand, 100 ng/ml recombinant human IL-11, 10 ng/ml recombinant murine IL-3 (PeproTech, Germany), 10% fetal calf serum, 1% penicillin/streptomycin, and 2 mmol/l glutamine (here after refer as I10-S3F11 medium) at a density of 5×10^5^ cells/ml. The cells were transduced on days 3, 4, 5, and 6 using 10^5^ cells and an MOI of 10–30 per transduction. For each vector or vector dose, three replicate transductions were performed in three different wells of a 24 well tissue culture plate and vector copy number for each well is separately measured later on. Virus preloading was carried out on RetroNectin-coated (TaKaRa, Japan) suspension culture dishes by spinoculation for 30 minutes at 4°C. The transduction starts with 1×10^5^ cells seeded into 500 µl medium and was increased by 250 µl medium on the following days, so that the culture volume was 1.25 ml on day 6. On day 10 (four days after the final addition of virus), an aliquot of cells were taken and treated with Benzonase Nuclease at a concentration of 50 units/ml for one hour at 37 degree. Genomic DNA was then extracted from the cells for vector copy number measurement using quantitative PCR. After retroviral transduction, the BM cells were expanded as mass cultures for 2 weeks in the I10-S3F11 medium. Cell concentration was maintained below 2×10^6^/ml by changing medium every 2–3 days. After mass culture expansion for 14 days the BM cells from each transduction were plated into a single 96-well plate at a density of 100 cells/well. Cells from the SFFV-GFP vector group at the highest dose were also plated at 10 cells/well into a 96-well plate. Two weeks later the wells with active cell growth were counted.

## Supporting Information

Figure S1 **Immune reconstitution in secondary recipient mice (peripheral blood).** Bone marrow cells from γc^−/−^ mice were transduced with the vectors indicated and transplanted into γc^−/−^Rag2^−/−^ mice. 5 months later, bone marrow cells were harvested and transplanted into secondary γc^−/−^Rag2^−/−^ mice. 18 weeks later, CD4+, CD8+ and B220+ cell counts in the peripheral blood were obtained using flow cytometry.
(DOCX)

Figure S2 **Immune reconstitution in spleens from secondary recipient mice.** Bone marrow cells from γc^−/−^ mice were transduced with the vectors and transplanted into γc^−/−^Rag2^−/−^ mice. 5 months later, bone marrow cells were harvested and transplanted into secondary γc^−/−^Rag2^−/−^ mice. 18 weeks later, percentage of CD4+, CD8+, B220+ and NK1.1+ cells in the spleen were analyzed by flow cytometry.
(DOCX)

Figure S3 **Peripheral blood cell counts in primary transplant recipients at 30 weeks (Exp#2).**
(DOCX)

Figure S4 **Blood chemistry results in primary transplant recipients at 30 weeks (Exp#2).**
(DOCX)

Figure S5 **Vector insertion in Mecom gene in leukemia #925 (Exp#2).** One of the SFFV vector insertion sites in leukemia #925 was identified using inverse-PCR. It is located in a intron of the MDS1 and EVI1 complex locus protein EVI1 (Mecom), in the reverse orientation relative to the Mecom gene.
(DOCX)

Figure S6 **Result of myeloid immortalization assay.** In order to be more objective about the counting of wells with cell growth, we also measured cell proliferation and viability by using a TACS MTT Cell Proliferation assay (Trevigen). Briefly, two weeks after culture in 96 well plates, 10 ul MTT reagent was added into each well. The plates were incubated for 2.5 hours at 37 degree. 100 ul detergent reagent was then added into each well and the plates were kept in the dark at room temperature for overnight. The absorbance at 562 nm was measured in a BioTek Synergy 2 plate reader.
(DOCX)

Table S1 **Tumor characteristics in secondary recipients (Exp1 and Exp2).**
(DOCX)
