# Supplementary material for: Mouse Transplant Models for Evaluating the Oncogenic Risk of a Self-Inactivating XSCID Lentiviral Vector
Source: PLoS One. 2013 Apr 23;8(4):e62333. doi: 10.1371/journal.pone.0062333 (PMC3633865; doi:10.1371/journal.pone.0062333)
Supplement: Figure S1 — Immune reconstitution in secondary recipient mice (peripheral blood). Bone marrow cells from γc−/− mice were transduced with the vectors indicated and transplanted into γc−/−Rag2−/− mice. 5 months later, bone marrow cells were harvested and transplanted into secondary γc−/−Rag2−/− mice. 18 weeks later, CD4+, CD8+ and B220+ cell counts in the peripheral blood were obtained using flow cytometry. (DOCX) [file pone.0062333.s001.docx]

**Figure S1: Immune reconstitution in secondary recipient mice (peripheral blood)**


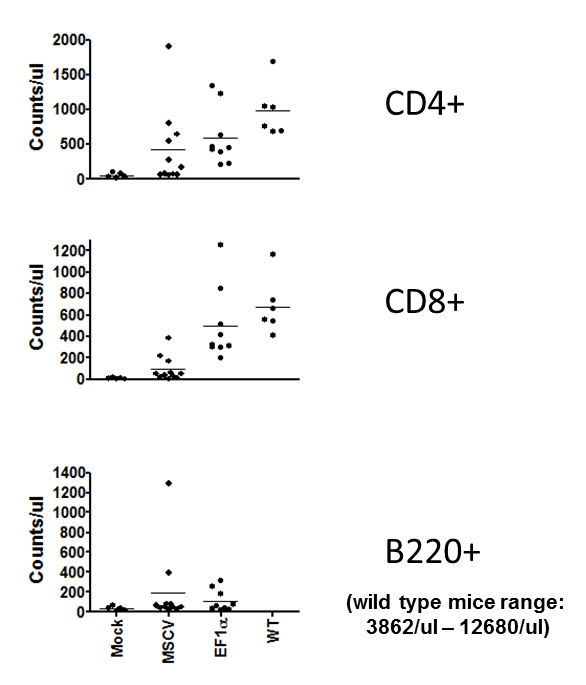


Figure S1. Bone marrow cells from γc^-/-^ mice were transduced with the vectors indicated and transplanted into γc^-/-^Rag2^-/-^ mice. 5 months later, bone marrow cells were harvested and transplanted into secondary γc^-/-^Rag2^-/-^ mice. 18 weeks later, CD4+, CD8+ and B220+ cell counts in the peripheral blood were obtained using flow cytometry.
