# Supplementary material for: Mouse Transplant Models for Evaluating the Oncogenic Risk of a Self-Inactivating XSCID Lentiviral Vector
Source: PLoS One. 2013 Apr 23;8(4):e62333. doi: 10.1371/journal.pone.0062333 (PMC3633865; doi:10.1371/journal.pone.0062333)
Supplement: Figure S2 — Immune reconstitution in spleens from secondary recipient mice. Bone marrow cells from γc−/− mice were transduced with the vectors and transplanted into γc−/−Rag2−/− mice. 5 months later, bone marrow cells were harvested and transplanted into secondary γc−/−Rag2−/− mice. 18 weeks later, percentage of CD4+, CD8+, B220+ and NK1.1+ cells in the spleen were analyzed by flow cytometry. (DOCX) [file pone.0062333.s002.docx]

**Figure S2: Immune reconstitution in spleens from secondary recipient mice**


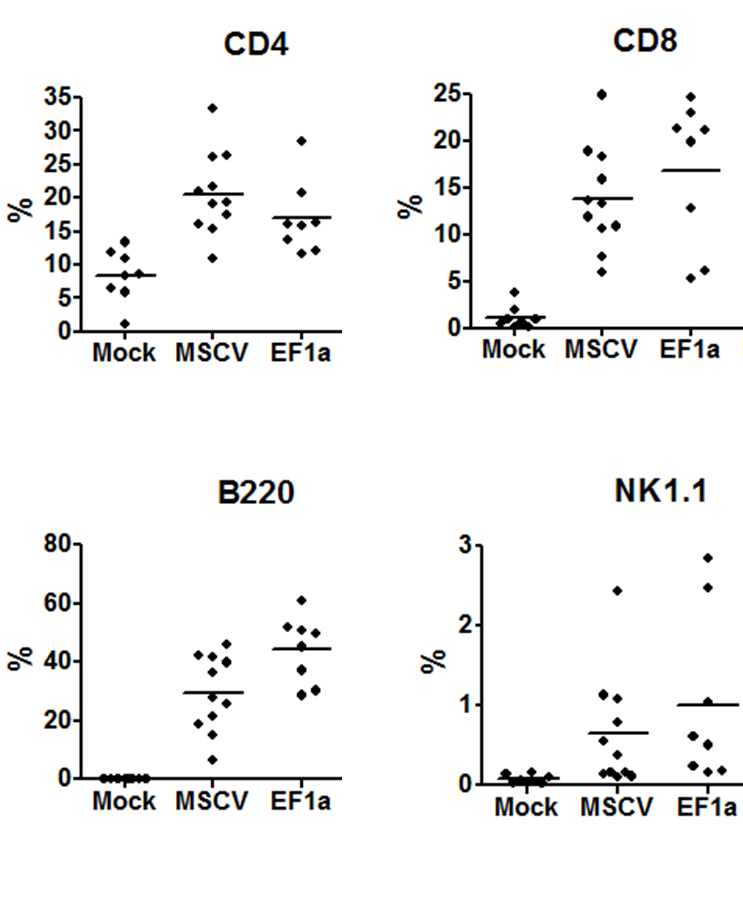


Figure S2. Bone marrow cells from γc^-/-^ mice were transduced with the vectors and transplanted into γc^-/-^Rag2^-/-^ mice. 5 months later, bone marrow cells were harvested and transplanted into secondary γc^-/-^Rag2^-/-^ mice. 18 weeks later, percentage of CD4+, CD8+, B220+ and NK1.1+ cells in the spleen were analyzed by flow cytometry.
