# Supplementary figures and images for: Mouse Transplant Models for Evaluating the Oncogenic Risk of a Self-Inactivating XSCID Lentiviral Vector
Source: PLoS One. 2013 Apr 23;8(4):e62333. doi: 10.1371/journal.pone.0062333 (PMC3633865; doi:10.1371/journal.pone.0062333)

**Figure S3. Peripheral blood cell counts in primary transplant recipients at 30 weeks (Exp#2)**


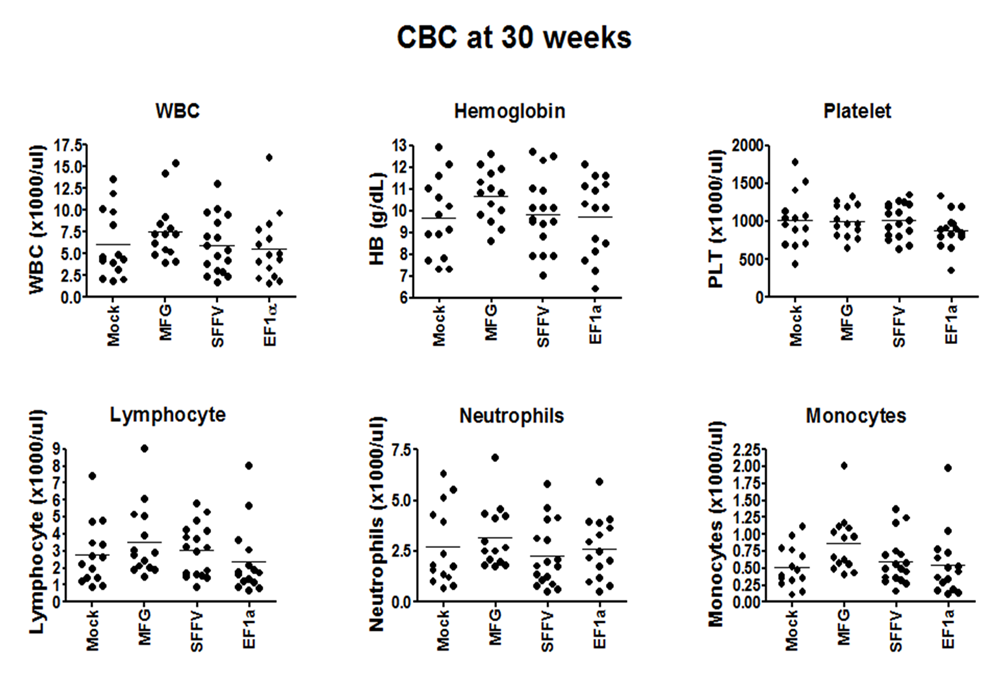

Supplement: Figure S3 — Peripheral blood cell counts in primary transplant recipients at 30 weeks (Exp#2). (DOCX) [file pone.0062333.s003.docx]

**Figure S4: Blood chemistry results in primary transplant recipients at 30 weeks (Exp#2)**


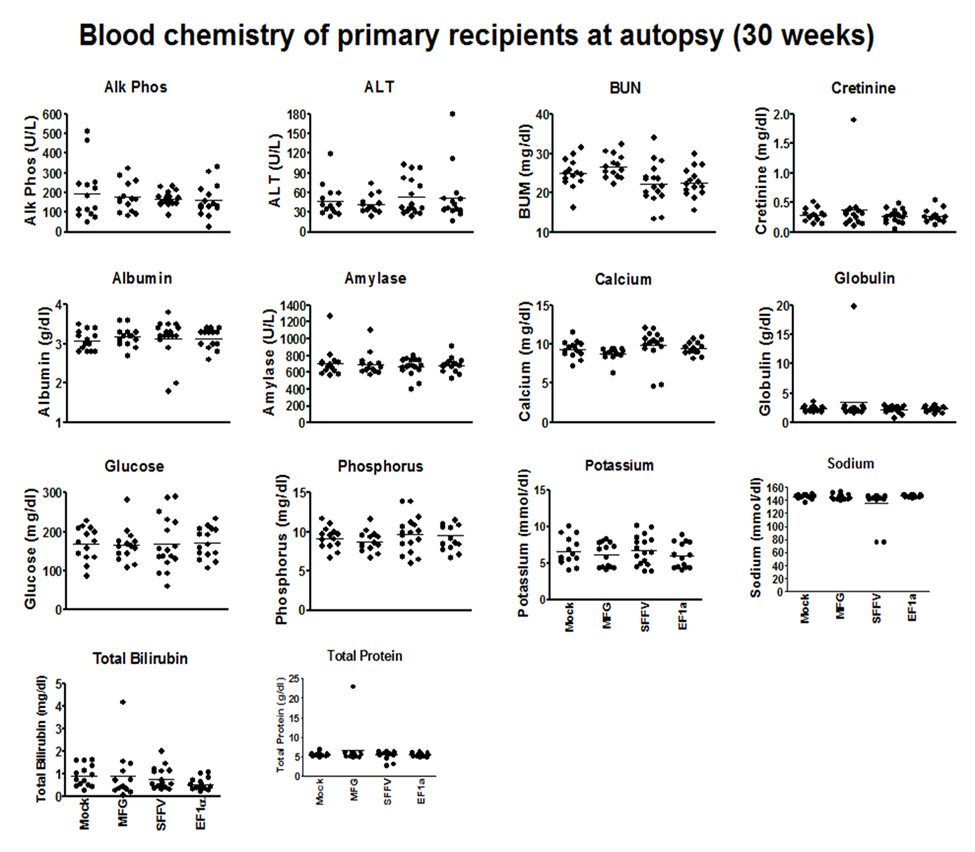

Supplement: Figure S4 — Blood chemistry results in primary transplant recipients at 30 weeks (Exp#2). (DOCX) [file pone.0062333.s004.docx]
