# Supplementary material for: Mouse Transplant Models for Evaluating the Oncogenic Risk of a Self-Inactivating XSCID Lentiviral Vector
Source: PLoS One. 2013 Apr 23;8(4):e62333. doi: 10.1371/journal.pone.0062333 (PMC3633865; doi:10.1371/journal.pone.0062333)
Supplement: Figure S5 — Vector insertion in Mecom gene in leukemia #925 (Exp#2). One of the SFFV vector insertion sites in leukemia #925 was identified using inverse-PCR. It is located in a intron of the MDS1 and EVI1 complex locus protein EVI1 (Mecom), in the reverse orientation relative to the Mecom gene. (DOCX) [file pone.0062333.s005.docx]

**Figure S5: Vector insertion in Mecom gene in leukemia #925 (Exp#2)**

Chr3: 30,126,082


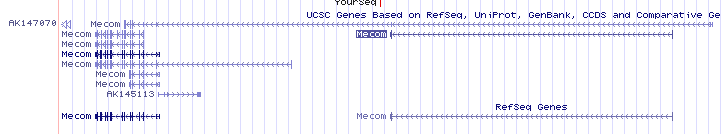


One of the SFFV vector insertion sites in leukemia #925 was identified using inverse-PCR. It is located in a intron of the MDS1 and EVI1 complex locus protein EVI1 (Mecom), in the reverse orientation relative to the Mecom gene.
