# Supplementary material for: Mouse Transplant Models for Evaluating the Oncogenic Risk of a Self-Inactivating XSCID Lentiviral Vector
Source: PLoS One. 2013 Apr 23;8(4):e62333. doi: 10.1371/journal.pone.0062333 (PMC3633865; doi:10.1371/journal.pone.0062333)
Supplement: Figure S6 — Result of myeloid immortalization assay. In order to be more objective about the counting of wells with cell growth, we also measured cell proliferation and viability by using a TACS MTT Cell Proliferation assay (Trevigen). Briefly, two weeks after culture in 96 well plates, 10 ul MTT reagent was added into each well. The plates were incubated for 2.5 hours at 37 degree. 100 ul detergent reagent was then added into each well and the plates were kept in the dark at room temperature for overnight. The absorbance at 562 nm was measured in a BioTek Synergy 2 plate reader. (DOCX) [file pone.0062333.s006.docx]

**Figure S6: Result of myeloid immortalization assay.**


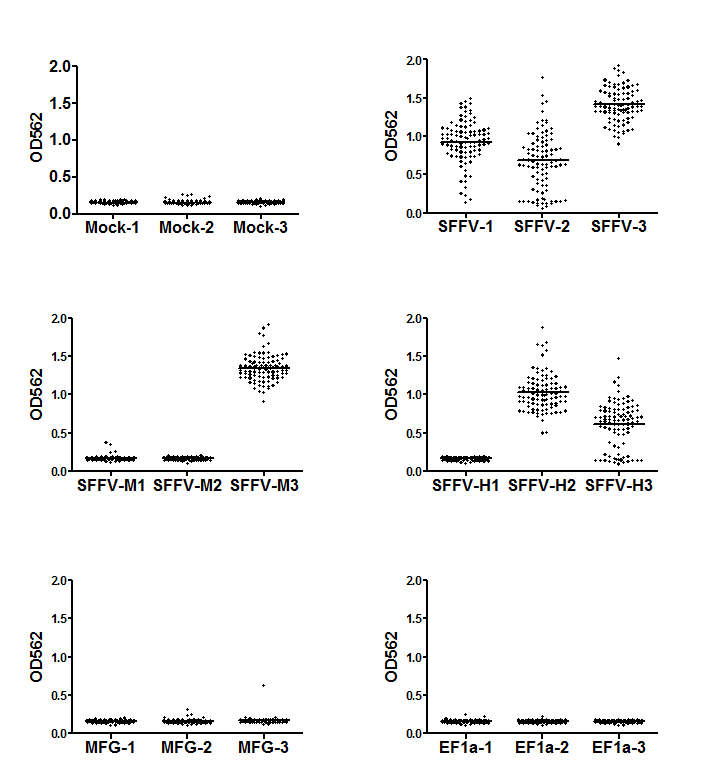


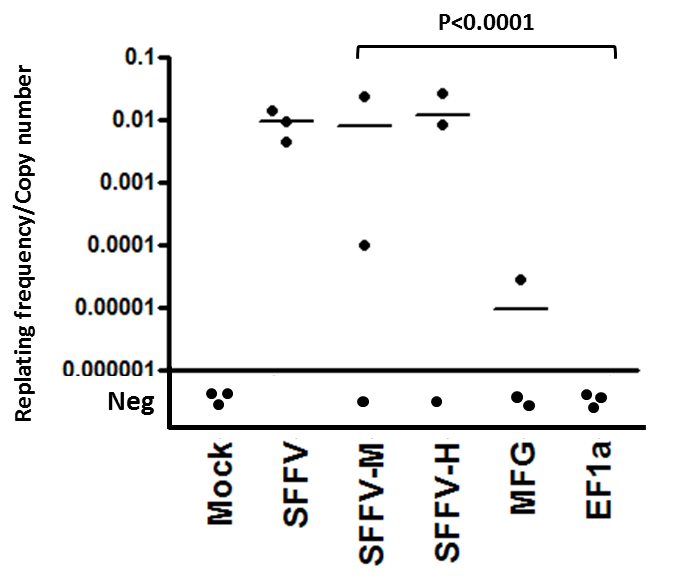


In order to be more objective about the counting of wells with cell growth, we also measured cell proliferation and viability by using a TACS MTT Cell Proliferation assay (Trevigen). Briefly, two weeks after culture in 96 well plates, 10ul MTT reagent was added into each well. The plates were incubated for 2.5 hours at 37 degree. 100ul detergent reagent was then added into each well and the plates were kept in the dark at room temperature for overnight. The absorbance at 562nm was measured in a BioTek Synergy 2 plate reader.
