# Supplementary material for: Mouse Transplant Models for Evaluating the Oncogenic Risk of a Self-Inactivating XSCID Lentiviral Vector
Source: PLoS One. 2013 Apr 23;8(4):e62333. doi: 10.1371/journal.pone.0062333 (PMC3633865; doi:10.1371/journal.pone.0062333)
Supplement: Table S1 — Tumor characteristics in secondary recipients (Exp1 and Exp2). (DOCX) [file pone.0062333.s007.docx]

Table S1. Tumor characteristics in secondary recipients (Exp1 and Exp2)

|  | Mouse ID | Time of diagnosis (weeks) | Diagnosis | Spleen weight (g) | CBC (x10^3^/ul) | Phenotype | Origin |
| --- | --- | --- | --- | --- | --- | --- | --- |
| **Mock** | 77 | 22 | mediastinal lymphoma | 0.039 | N/A | CD45.1-, CD4+, CD8+ | Recipient |
|  | 973 | 15 | T-cell lymphoma | normal | 3.84 | N/A | N/A |
|  | 726 | 28 | T-cell leukemia | 0.046 | 4.58 | N/A | N/A |
| **EF1a** | 203 | 25 | B-cell lymphoma | 1.161 | 10.1 | IgM+, B220+/- | Donor |
|  | 204 | 28 | B-cell lymphoma | 0.349 | 2.4 | IgM+, B220+/- | Donor |
|  | 205 | 27 | B-cell lymphoma | 0.193 | 4.9 | IgM+, B220+/- | Donor |
|  | 910 | 18 | T-cell lymphoma | 0.652 | 4.82 | CD45.2+, CD45.1-, CD4+, CD8+ | Recipient |
|  | 714 | 24 | T-cell Leukemia | 0.206 | 2.56 | CD45.2+, CD45.1-, CD4+, CD8+ | Recipient |
|  | 756 | 28 | T-cell Leukemia | 0.219 | 7.18 | CD45.2+, CD45.1-, CD4+, CD8+ | Recipient |
| **MFG** | 913 | 28 | Thymic lymphoma | 0.074 | 7.46 | N/A | N/A |
| **SFFV** | 637 | 19 | Myeloid leukemia | 0.514 | 167 | DsRed+, Mac1+, Gr1+ | Donor |
|  | 638 | 22 | Myeloid leukemia | 0.482 | 280.2 | DsRed+, Mac1+, Gr1+ | Donor |
|  | 639 | 26 | Myeloid leukemia | 0.526 | 182.4 | DsRed+, Mac1+, Gr1+ | Donor |
|  | 925 | 28 | Myeloid leukemia | 0.262 | 46.02 | DsRed+, Mac1+, Gr1+ | Donor |
|  | 927 | 28 | Myeloid leukemia | 0.209 | 24.44 | DsRed+, Mac1+, Gr1+ | Donor |
|  | 964 | 27 | B-cell leukemia | 0.479 | 54.34 | DsRed+B220+ | Donor |
|  | 965 | 28 | B-cell Leukemia | 0.272 | 15.58 | DsRed+B220+ | Donor |
|  | 959 | 22 | T-cell Leukemia | 0.277 | 25.92 | DsRed-, CD4+, CD8+ | Recipient |

Note: #203, #204 and #205 receive bone marrow from the same donor #364 (Exp 1); #637, 638 and 639 received bone marrow from the same donor #8046 (Exp2); #925, #927 received bone marrow cells from the same donor #8049(Exp2); #964, #965 received bone marrow cells from the same donor #8059(Exp2).
